# METTL3 Is Involved in the Development of Graves’ Disease by Inducing SOCS mRNA m6A Modification

**DOI:** 10.3389/fendo.2021.666393

**Published:** 2021-09-20

**Authors:** Rong-hua Song, Peng Du, Chao-qun Gao, Xue-rong Liu, Jin-an Zhang

**Affiliations:** Department of Endocrinology & Rheumatology, Shanghai University of Medicine & Health Sciences Affiliated Zhoupu Hospital, Shanghai, China

**Keywords:** Graves’ disease (GD), RNA modification, RNA methyltransferase, methyltransferase like 3 (METTL3), suppressor of cytokine signaling (SOCS)

## Abstract

**Objective:**

Epigenetic modifications in RNA are known to play critical roles in cell differentiation through regulating expressions of some key genes including members of the suppressor of cytokine signaling (SOCS) family. The present study aimed to unveil the relationship of SOCS mRNA methylation induced by methyltransferase like 3 (METTL3) with Graves’ disease (GD).

**Methods:**

Differently expressed genes (DEG) in GD tissues were identified using microarray analysis and further validated using CD4^+^ T cell microarray of GD tissues and isolated peripheral blood mononuclear cells (PBMCs). Furthermore, expressions of METTL3 targeted genes were detected using METTL3 knock-down experiment in RAW264.7 cells.

**Results:**

High throughput microarrays revealed that METTL3 and SOCS molecules were aberrantly expressed in thyroid tissues and CD4^+^T cells of GD compared to the controls. Bioinformatic analysis was undertaken by searching databases of found genes of the SOCS family that possessed many mRNA m6A modification loci. METTL3 knock-down experiment revealed that expressions of SOCS family members SOCS1, SOCS2, SOCS4, SOCS5, and SOCS6 were increased after METTL3 knock-down.

**Conclusions:**

For the first time, the present study revealed the relationship between m6A modification and GD and indicated that METTL3 may be involved in the development of GD by inducing mRNA m6A methylation modification of SOCS family members.

## Introduction

Graves’ Disease (GD) is the main clinical sub-type of autoimmune thyroid diseases (AITDs), leading to a decline in quality of life and increasing the risk of death, and thus threatening public health. It is the cause of 90% hyperthyroidism, annually affecting 20-50 in 100,000 people, mainly aged between 30-50 years old, among whom, 5% were females and only 0.5% were males (1, 2). In GD patients, thyrotropin-receptor antibody (TRAb) imitates the role of TSH and stimulates thyroid follicular epithelial cells to produce and secret hormones, thus inducing thyrotoxicosis. As of now, no therapy targets the etiology of GD, instead, all tend to focus on the treatment of basic symptoms (3). GD is an autoimmune disease caused by both genetic and environmental factors. Thus far, genetic-associated studies have identified mainly thyroglobulin (Tg), thyrotropin receptor (TSHR), PTPN22, IL-17, and IL-22 as the susceptible genes of GD (4–6). Similarly, immune dysfunction caused by the imbalance of immune cells was also found to play an important role in the pathogenesis of GD. Additionally, our previous studies, along with others, have also revealed that epigenetic factors play a pivotal part in the etiology of GD (7). Yet, despite these findings, the specific molecular mechanism underlying the above-mentioned multiple factors involved in the pathogenesis of GD has not been clarified.

N^6^-methyladenosine (m^6^A) is the most abundant and stable form of mRNA modification in most species and is also an important epigenetic marker involved in all aspects of the life process. mRNA m^6^A modification has been reported to influence mRNA alternative splicing, translation, and stability, thereby regulating the expression and function of targeted genes (8). Several studies have revealed that mRNA m^6^A modification is associated with the differentiation and immune response of immune cells (9–12). Furthermore, the recent identification of key enzymes for m^6^A modification including m^6^A methyltransferase (writer), m^6^A demethylase (eraser), and m^6^A RNA binding protein (reader) has dramatically deepened our understanding of RNA epigenesis (13, 14). Among these enzymes, methyltransferase like 3 (METTL3) is the key methylation modification enzyme. It has been evidenced that METTL3 is involved in multiple life processes by regulating mRNA level and the protein expression of some key molecules (15). Moreover, some studies found that polymorphisms of METTL3 and fat mass and obesity associated (FTO) genes are involved in the pathogenesis of some autoimmune diseases, such as rheumatoid arthritis (RA) (16) and latent autoimmune diabetes (LADA) (17). Our recent study also uncovered that METTL3 polymorphisms are involved in the pathogenesis of AITDs, including GD (18). However, the mechanism underlying the involvement of mRNA m^6^A modification in GD development is still incompletely understood.

mRNA m^6^A methylation alters mRNA levels and the subsequent protein expression of targeted genes by influencing mRNA stability and degradation rate. This mechanism is extremely important in regulating the expression of immediate-early genes (IEGs) (19). Among these IEGs, the suppressors of the cytokine signaling (SOCS) family, which mainly consist of one CIS and 7 SOCS proteins, are involved in many biological processes, such as cell proliferation, differentiation, and signal transduction. SOCS proteins also play a pivotal role in regulating the immune system and the development of immune-mediated diseases (20). Studies have revealed aberrant SOCS1 and SOCS3 expressions in diverse autoimmune diseases (21). However, whether METTL3 is involved in GD by inducing the methylation modification of the SOCS family has not been elucidated. Therefore, to explore the role of METTL3 in GD development and better understand the potential pathomechanism of GD, we analyzed our previous microarray data using bioinformatic analysis tools, validated changes in mRNA levels of METTL3 and SOCS family members using real-time quantitative polymerase chain reaction (qPCR) technology in clinical samples, and performed functional analyses in cultured cells by knocking-down METTL3.

## Materials and Methods

### Microarray and Bioinformatic Analyses

First, we screened the differentially expressed genes (DEGs) in our previously published GD microarray, which included 4 GD cases and 3 controls (22). In addition, to further explore the possible functions of these DEGs, by using the online functional annotation tools in the Database for Annotation, Visualization and Integrated Discovery (DAVID, http://david.abcc.ncifcrf.gov/) (23, 24), we analyzed the functional pathways by the Gene Ontology (GO) functional enrichment and Kyoto Encyclopedia of Genes and Genomes (KEGG) pathway methods. Second, we searched the Gene Expression Omnibus (GEO) (https://www.ncbi.nlm.nih.gov/geo/) and found a genome-wide gene expression dataset, GSE9340, which includes 18 GD thyroid tissues. Adopting the same analysis, we screened the DEGs from GSE9340. We combined DEGs from our microarray data and GSE9340, and carried out the gene co-expression network bioinformatics analysis by using the WGCNA R software package as described previously (25). Finally, we further identified the potential functional pathways of genes in key co-expression modules using the DAVID database including GO and KEGG pathways.

### Validation for Selected Genes

To further validate the expression of METTL3 and SOCS family members, we first explored Dataset GSE71956, which provides a genome-wide gene expression profile in CD4^+^ T cells from 15 GD patients and 10 controls. Furthermore, we isolated peripheral blood mononuclear cells (PBMCs) from whole blood samples of 26 newly recruited GD patients and 26 age- and sex-matched healthy volunteers and extracted total RNA from these PBMCs samples. The TRAb were positive for all the GD patients. For the healthy controls, we chose those who had normal thyroid function and negative TRAb, anti-thyroglobulin antibody (TgAb), and anti-thyroid peroxidase antibody (TPOAb). Those with thyroid diseases or other autoimmune diseases were excluded from the control group. After qualification and quantification by Nano Drop 2.0, we examined mRNA levels of METTL3, METTL14, FTO, and ALKBH5 using real-time qPCR with primers GTGATCGTAGCTGAGGTTCGT and GGGTTGCACATTGTGTGGTC for METTL3, TGGACCTTGGAAGAGTGTGTTT, and CATGAGGCAGTGTTCCTTTGTT for METTL14, TTGCCCGAACATTACCTGCT, and TGTGAGGTCAAAAAAGCGCAGAG for FTO, TCAAGCCTATTCGGGTGTCG, and AGCAGCATATCCACTGAGCA for ALKBH5, respectively.

### Effects of the Knockdown of METTL3 on the Expression of SOCS Family Members

To explore the function of METTL3, we knocked down METTL3 in a mouse monocyte macrophage leukemic cell line RAW264.7 by transfecting METTL3 shRNA with METTL3 shRNA. The METTL3 shRNA sequences were designed as follows: upper-GATCCGGTTCGTTCCACCAGTCATAATTCAAGAGATTATGACTGGTGGAACGAACCTTTTTTG, lower-AATTCAAAAAAGGTTCGTTCCACCAGTCATAATCTCTTGAATTATGACTGGTGGAACGAACCG. Using the real-time PCR method, we identified that METTL3 gene expression was largely down-expressed after the interfering. Then we detected the gene expression of SOCS family members, such as SOCS1, SOCS2, SOCS3, SOCS4, SOCS5, SOCS6, and SOCS7, by real-time PCR. The primers of these genes are shown below:

SOCS1 former: GACGCCTGCGGCTTCTATT,
reverse: CAGCTCGAAAAGGCAGTCG;
SOCS2 former: ACGGAATGGGACTGTTCACC,
reverse: AAGGCAGTCCCCAGATCGTA;
SOCS3 former: TGGTCACCCACAGCAAGTTT,
reverse: TCGCTTTTGGAGCTGAAGGT;
SOCS4 former: CGGAGTCGAAGTGCTGACAG,
reverse: ACTCAATGGACGAACAGCTAAG;
SOCS5 former: TTCCCATGAGAACTTACAGCAAG,
reverse: TTTTGTGCTAAATCCGAGCCA;
SOCS6 former: AAGCAAAGACGAAACTGAGTTCA,
reverse: CAGCTCCCGAATAAAGAGTCATC;
SOCS7 former: GAAACCCAGGTTGACAAGAACT,
reverse: TCCACAAGCGATACTGTCTCA.

### Statistical Analysis

SPSS 20.0 software (IBM, Chicago, USA) was used to carry out the statistical analysis. A T-test was performed to analyze the differences in gene expression between two groups. WGCNA R software package was used to find functional pathways including DEGs. p value smaller than 0.05 was considered statistically significant.

## Results

### mRNA Expressions Profile in GD

**Figure 1:**
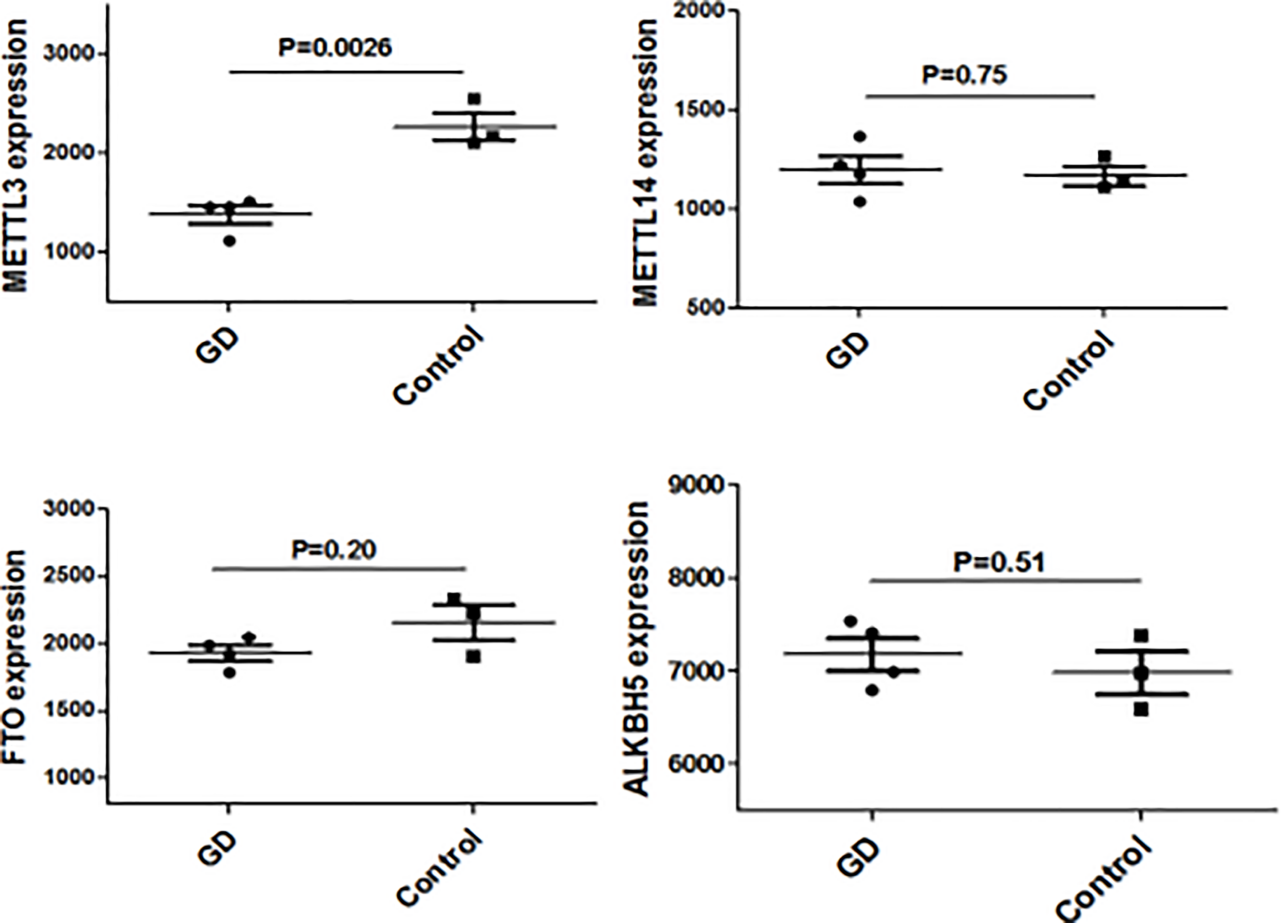
METTL3 mRNA expressions in thyroid from GD and controls.

**Figure 2:**
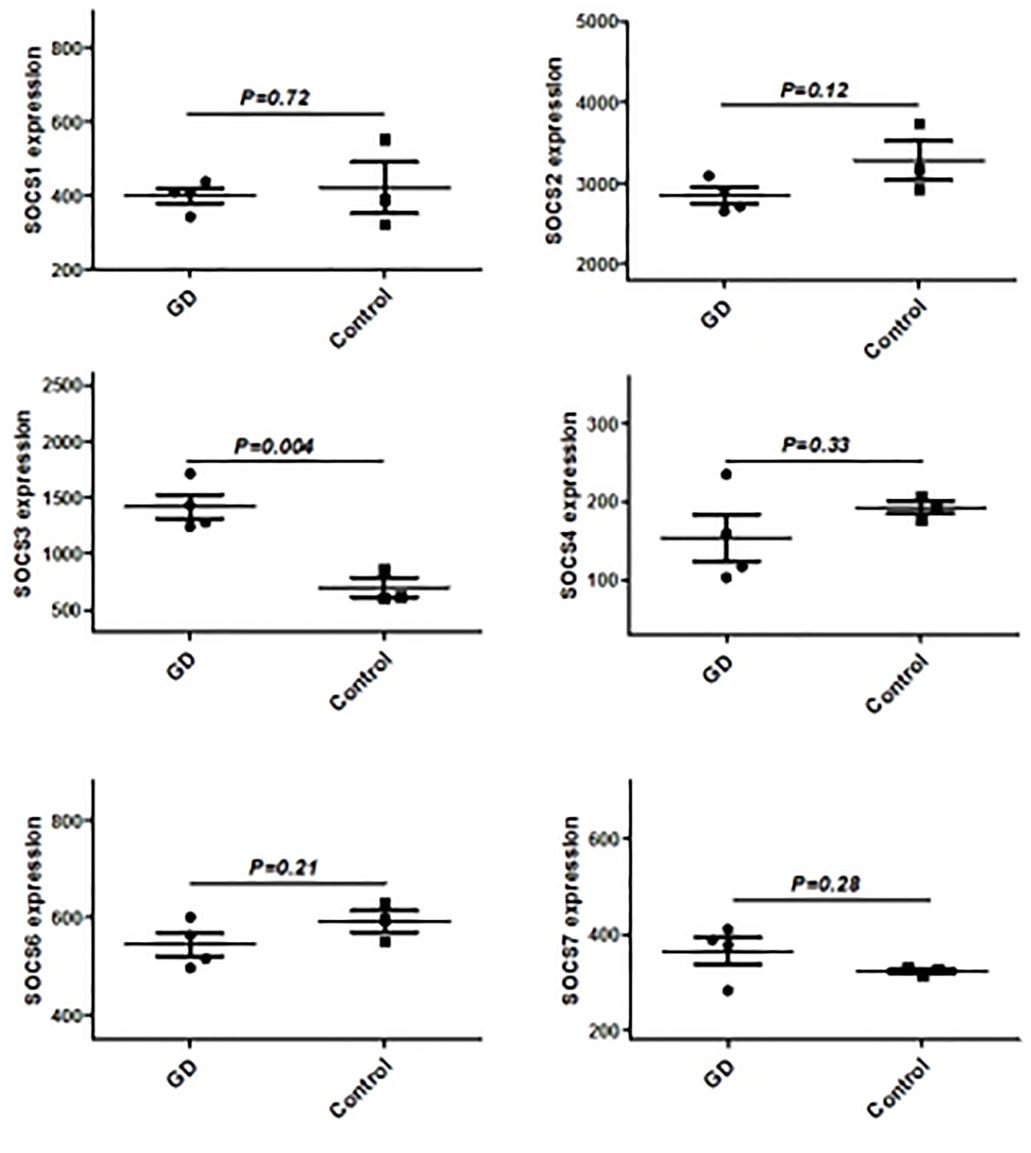
The mRNA expression of SOCS family main members in in thyroid from GD and controls.

**Figure 3:**
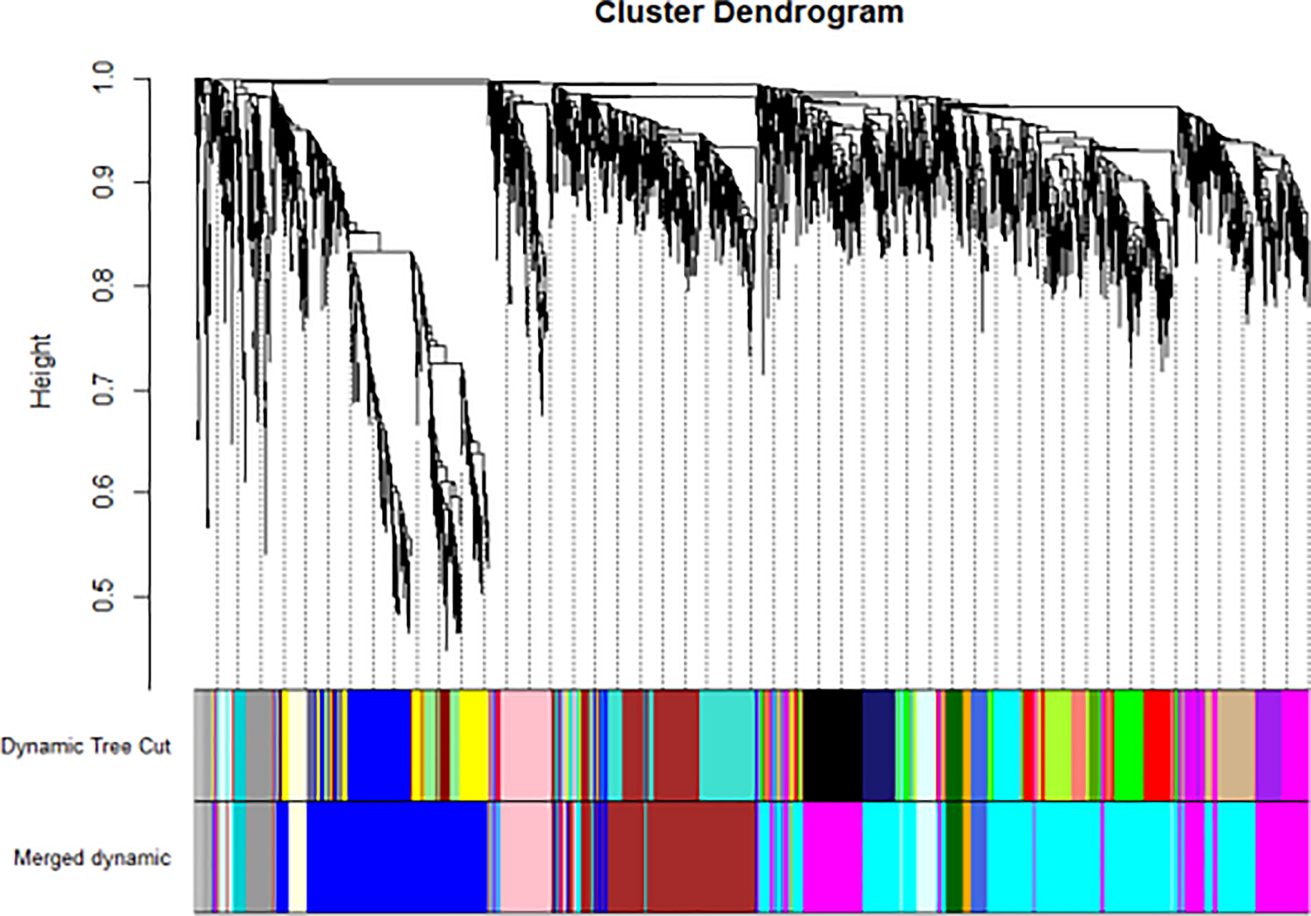
WGCNA found several GD related key gene expression modules (The most significant is the black module).

We have previously isolated 7 thyroid tissues from recruited 4 GD patients and 4 controls, screened the microRNA/mRNA profile by gene microarray, and found more than 2800 aberrant expressed mRNAs related to GD (22). Through GO and KEGG pathway analysis, we found that these aberrantly expressed mRNAs were enriched in the differentiation and regulation of immune cells (22). Among these genes, METTL3 and its target genes, SOCS family members, were all included. METTL3 mRNA level was significantly lower in GD thyroid tissues than in normal thyroid tissues (P=0.0026, **Figure 1**) while SOCS3 mRNA level was significantly higher in GD thyroid samples than in normal thyroid samples (P=0.04, **Figure 2**). Moreover, we combined DEGs in thyroid tissues from our precious microarray data and GSE9340 microarray data downloaded from the GEO database and created several GD related gene co-expression modules. Some of these modules were associated with the onset of GD (**Figure 3**), including a blue module composed of 553 genes and a green module composed of 903 genes. Among the 903 genes in the green module are key genes for m^6^A methylation, such as METTL3. GO function enrichment analysis indicated that the genes in the blue module are mainly involved in immune response pathways such as innate immune response (Bonferoni P = 3.18E^-23^) and adapted immune response (Bonferoni P = 2.92E^-20^) pathways (**Table 1**) and the genes in the green module are mainly correlated with RNA machinery and metabolism pathways (**Table 2**), implying that mRNA methylation plays vital roles in the etiology of GD.

**Table 1:** GD related key gene co-expression modules---blue module gene function riched GO pathway.

Pathway ID	Number of gene	Name of pathways	Bonferoni P-value
GO:0006955	165	Immune response	1.14E-49
GO:0002376	195	Immune system process	2.36E-44
GO:0002682	129	Regulation of immune system process	4.65E-34
GO:0006952	140	Defense response	4.68E-34
GO:0050776	98	Regulation of immune response	1.51E-29
GO:0002684	99	Positive regulation of immune system process	9.93E-27
GO:0045321	95	Leukocyte activation	2.88E-26
GO:0045087	90	Innate immune response	3.18E-23
GO:0050778	76	Positive regulation of immune response	3.40E-23
GO:0001775	101	Cell activation	1.33E-21
GO:0002250	58	Adaptive immune response	2.92E-20
GO:0007159	68	Leukocyte cell-cell adhesion	2.28E-18
GO:0070486	65	Leukocyte aggregation	1.14E-17
GO:0071593	64	Lymphocyte aggregation	1.93E-17
GO:0042110	63	T cell activation	6.80E-17

**Table 2:** GD related key gene co-expression modules---green module gene function riched GO pathway.

Pathway ID	Number of gene	Name of pathways	Bonferoni P-value
GO:0006396	85	RNA processing	4.02E-11
GO:0006613	33	Cotranslational protein targeting to membrane	2.48E-10
GO:0006614	31	SRP-dependent cotranslational protein targeting to membrane	2.36E-09
GO:0045047	31	Protein targeting to ER	2.36E-09
GO:0006612	37	Protein targeting to membrane	1.05E-08
GO:0072599	31	Establishment of protein localization to endoplasmic reticulum	1.47E-08
GO:0016071	64	mRNA metabolic process	1.52E-08
GO:0000184	32	Nuclear-transcribed mRNA catabolic process, nonsense-mediated decay	2.59E-08
GO:0003723	129	RNA binding	4.78E-08
GO:0005840	36	Ribosome	1.04E-07
GO:0022626	30	Cytosolic ribosome	2.19E-07
GO:0000956	37	Nuclear-transcribed mRNA catabolic process	3.99E-07
GO:0070972	32	Protein localization to endoplasmic reticulum	4.64E-07
GO:0006364	40	rRNA processing	5.33E-07
GO:0016072	40	rRNA metabolic process	5.33E-07
GO:0006402	37	mRNA catabolic process	1.16E-06
GO:0006413	39	Translational initiation	1.34E-06
GO:0003735	34	Structural constituent of ribosome	2.38E-06
GO:0015934	21	Large ribosomal subunit	3.10E-06
GO:0006396	85	RNA processing	4.02E-11
GO:0006613	33	Cotranslational protein targeting to membrane	2.48E-10
GO:0006614	31	SRP-dependent cotranslational protein targeting to membrane	2.36E-09

### Validation of Aberrant Genes in GD

**Figure 4:**
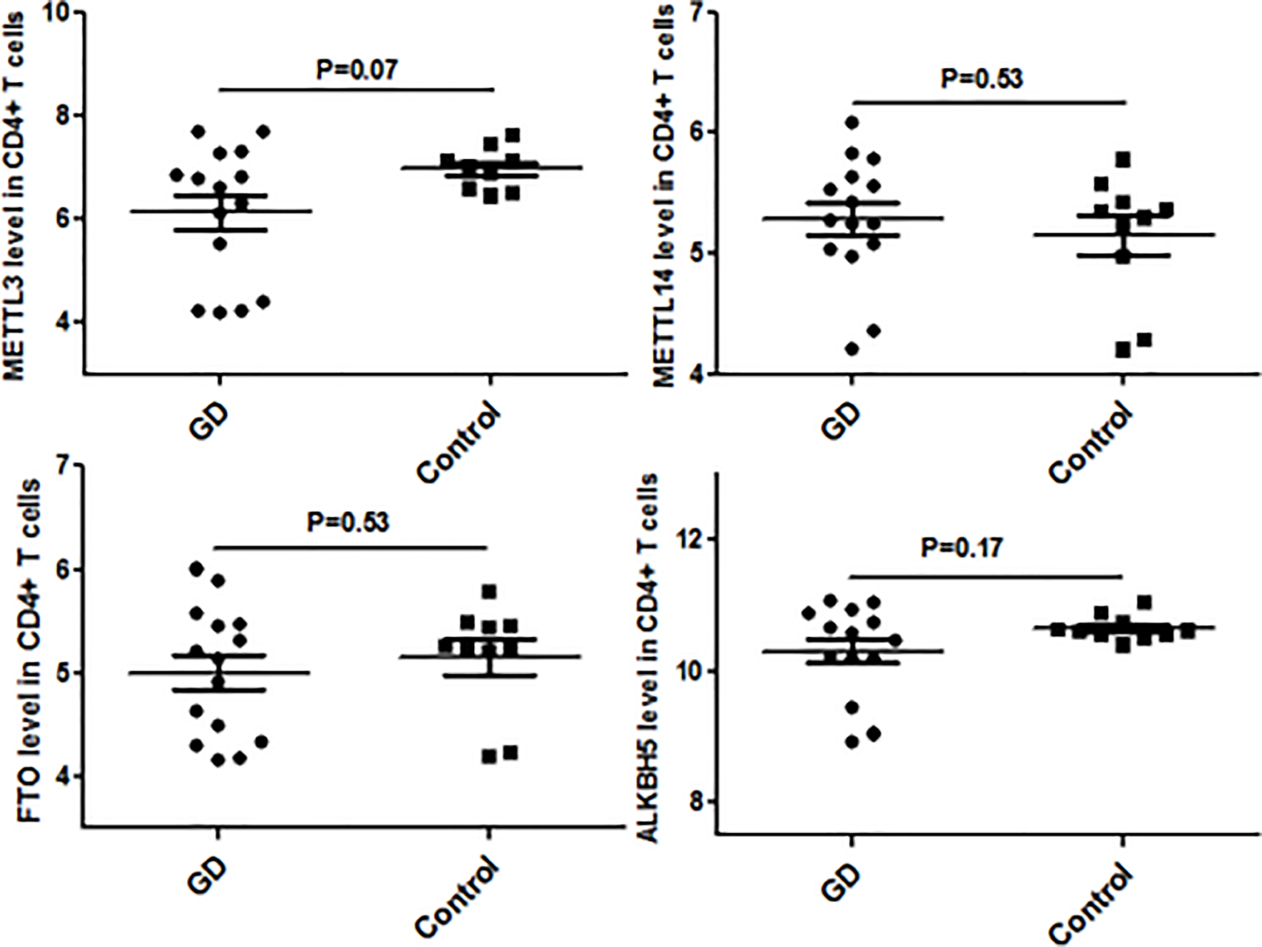
mRNA modification key enzymes’ expressions in CD4+T cells of GD.

Since CD4^+^ T cells play important roles in GD, we then detected the expression of some genes that are the key enzymes of mRNA m^6^A methylation including METTL3 by using GSE71956, a CD4^+^ T cell gene expression profile microarray dataset. As shown in **Figure 4**, METTL3 expression in CD4^+^ T cells was lower in GD than in controls, although it was not statistically significant. A similar trend was also observed in other key genes involved in mRNA m6A modification, such as FTO and ALKBH5. It would be worthwhile to explore these trends in further depth in the future.

We used bioinformatic database RMBase v2.0 (http://rna.sysu.edu.cn/rmbase/index.php), which targets mRNA m^6^A modification to screen m^6^A modification loci. The results revealed numerous m^6^A modification loci on mRNA of SOCS family members. **Table 3** displays those with motif score > 350 (motif score ranges from 0 to 500, the larger value represents higher accuracy).

**Table 3:** m6A modification loci in the mRNA of SOCS family members (main part).

SOCS members	Chromosome	ModID	Motif score	region
SOCS1	Chr16(11348442-11348443)	m6A_site_162250	369.74	3'UTR
SOCS1	Chr16(11348969-11348970)	m6A_site_162254	369.74	CDS
SOCS2	Chr12(93966490-93966491)	m6A_site_105936	419.59	5'UTR
SOCS2	Chr12(93966573-93966574)	m6A_site_105938	419.59	5'UTR
SOCS2	Chr12(93968677-93968678)	m6A_site_105949	419.59	CDS/3'UTR
SOCS2	Chr12(93968897-93968898)	m6A_site_105953	419.59	CDS/3'UTR
SOCS2	Chr12(93966560-93966561)	m6A_site_105937	371.87	5'UTR
SOCS2	Chr12(93968769-93968770)	m6A_site_105951	371.87	CDS/3'UTR
SOCS2	Chr12(93966736-93966737)	m6A_site_105940	369.74	CDS
SOCS3	Chr17(76353316-763533167)	m6A_site_206141	419.59	3'UTR
SOCS3	Chr17(76353537-76353538)	m6A_site_206143	419.59	3'UTR
SOCS3	Chr17(76354571-76354572)	m6A_site_206160	419.59	CDS
SOCS3	Chr17(76356144-76356145)	m6A_site_206173	419.59	5'UTR
SOCS3	Chr17(76353702-76353703)	m6A_site_206146	371.87	3'UTR
SOCS3	Chr17(76353887-76353888)	m6A_site_206148	371.87	3'UTR
SOCS3	Chr17(76354182-76354183)	m6A_site_206155	371.87	3'UTR
SOCS3	Chr17(76354370-76354371)	m6A_site_206158	371.87	3'UTR
SOCS3	Chr17(76355126-76355127)	m6A_site_206169	371.87	CDS
SOCS4	Chr14(55498662-55498663)	m6A_site_129198	419.59	5'UTR/Exon
SOCS4	Chr14(55509988-55509989)	m6A_site_129204	419.59	CDS
SOCS4	Chr14(55510908-55510909)	m6A_site_129222	419.59	CDS
SOCS4	Chr14(55494015-55494016)	m6A_site_129195	369.74	5'UTR/Exon
SOCS4	Chr14(55510855-55510856)	m6A_site_129220	369.74	CDS
SOCS5	Chr2(46926501-46926502)	m6A_site_259666	419.59	5'UTR
SOCS5	Chr2(46986112-46986113)	m6A_site_259676	419.59	CDS
SOCS5	Chr2(46986888-46986889)	m6A_site_259689	419.59	CDS
SOCS5	Chr2(46986903-46986904)	m6A_site_259690	419.59	CDS
SOCS6	Chr18(67992545-67992546)	m6A_site_216257	419.59	CDS
SOCS6	Chr18(67992559-67992560)	m6A_site_216258	419.59	CDS
SOCS6	Chr18(67992887-67992888)	m6A_site_216263	419.59	CDS
SOCS6	Chr18(67993294-67992546)	m6A_site_216271	419.59	CDS
SOCS7	Chr17(36508291-36508292)	m6A_site_192042	419.59	CDS
SOCS7	Chr17(36508629-36508630)	m6A_site_192046	419.59	CDS/Exon
SOCS7	Chr17(36555962-36555963)	m6A_site_192056	419.59	3'UTR
SOCS7	Chr17(36556240-36556241)	m6A_site_192060	419.59	3'UTR
CISH	Chr3(50644562-50644563)	m6A_site_325389	419.59	3'UTR/Exon
CISH	Chr3(50644971-50644972)	m6A_site_325396	419.59	3'UTR/Exon
CISH	Chr3(50645098-50645099)	m6A_site_325398	419.59	CDS/Exon
CISH	Chr3(50645392- 50645393)	m6A_site_325400	419.59	CDS/Exon

**Figure 5:**
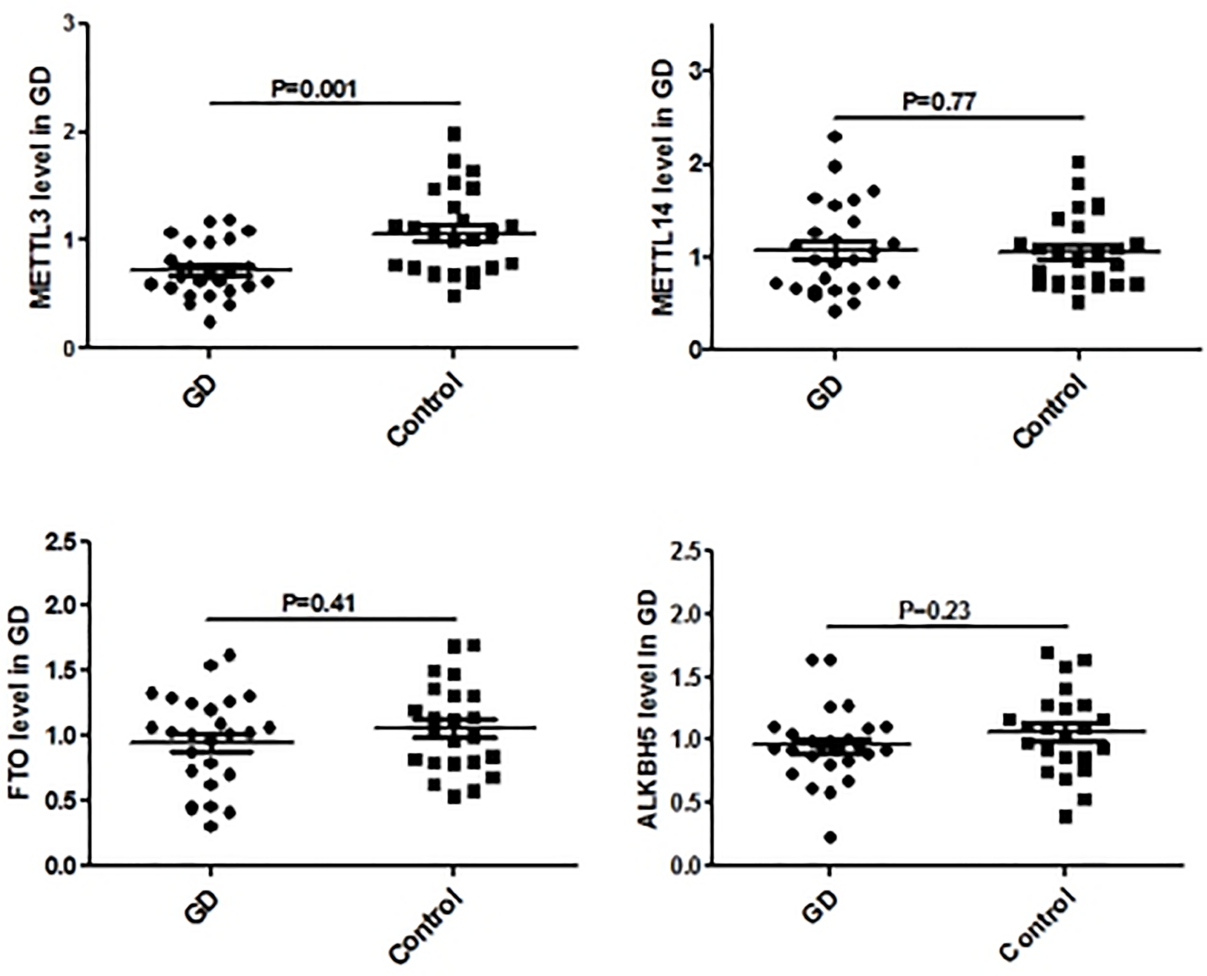
mRNA modification key enzymes’ expressions in PBMCs of GD.

**Figure 5** shows the real-time qPCR results on expressions of genes key to mRNA m6A methylation in PBMCs from clinical GD patients and healthy controls, indicating that METTL3 mRNA level was significantly lower in the PBMCs of GD patients than in healthy controls (P = 0.001). By contrast, mRNA levels of METTL14, FTO, and ALKBH5 were not significantly different among these two groups.

### Influence of METTL3 Knockdown on the Expression of SOCS Family Members

**Figure 6:**
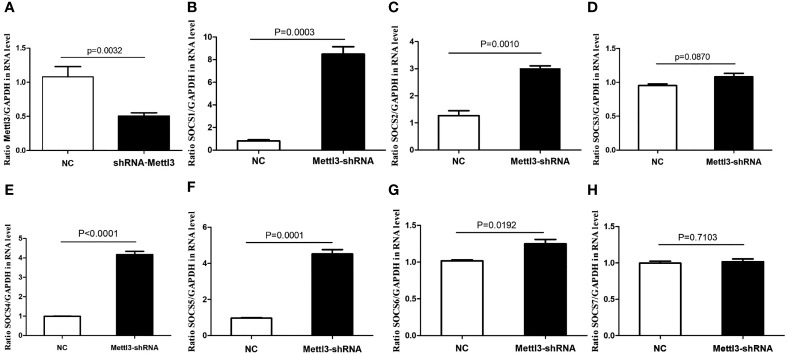
mRNA expressions of SOCS family members in METTL3 knock-down RAW 264.7 cells. **(A)** It showed the significant lower expression of METTL3 after METTL3 knock-down; **(B)** SOCS1 mRNA expression after METTL3 knock-down; **(C)** SOCS2 mRNA expression after METTL3 knock-down; **(D)** SOCS3 mRNA expression after METTL3 knock-down; **(E)** SOCS4 mRNA expression after METTL3 knock-down; **(F)** SOCS5 mRNA expression after METTL3 knock-down; **(G)** SOCS6 mRNA expression after METTL3 knock-down; **(H)** SOCS7 mRNA expression after METTL3 knock-down.

*In vitro* functional analysis found that the mRNA levels of SOCS1, SOCS2, SOCS4, SOCS5, and SOCS6 were significantly higher in METTL3 knockdown RAW264.7 cells (P<0.05), while SOCS3 mRNA level was not significantly changed after METTL3 knockdown (**Figure 6** **)**.

## Discussion

GD is a common autoimmune disease that is susceptible to multiple factors. Among them, genetic, environmental, and immune factors all exert important roles (26). Additionally, epigenetics factors, which play vital roles in integrating environmental and genetic elements, are closely related to the predisposition of GD (7). Currently, mRNA m^6^A modification as a form of epigenetic modification has become a new research hotspot in various kinds of diseases including autoimmune diseases (13, 14). As a key RNA methylation modification enzyme, METTL3 is reported to regulate the mRNA and protein levels of several key molecules and plays a key role in diverse life processes (15). Although we have previously shown that METTL3 polymorphisms confer risk for the susceptibility of GD (18) and mRNA m6A modification is known to regulate immune cell differentiation and immune response (10–12) and is involved in the development of some autoimmune diseases (16, 17). The mechanisms underlying the effects of m6A modification on autoimmune diseases including GD are still incompletely understood and require more in-depth studies. In this study, we analyzed previous genome-wide expression microarray data on GD and found that METTL3, a key enzyme for mRNA m^6^A modification was aberrantly downregulated in GD thyroid tissues. Furthermore, METTL3 mRNA levels were also decreased in the PBMCs of GD patients. These data indicate that METTL3 induced mRNA m^6^A modification is linked to the pathomechanism of GD.

WGCNA is a widely used data mining method in a biological system to explore disease-related gene co-expression modules from high throughput data. The gene co-expression module involves a cluster of genes that are intensely related in expression and function levels and play key roles in disease development. In this study, WGCNA analysis was used to reveal gene co-expression modules that are intensely related to GD and enriched in the signaling pathway of RNA translation and metabolism. The results showed that METTL3 is a key component of these modules, which indicates that mRNA m^6^A modification by METTL3 may be involved in the development of GD.

It is well known that epigenetic modifications in RNA exert critical roles in regulating cell differentiation through changing the expressions of some key genes, like SOCS family members (19–21). Therefore we searched the bioinformatic database and found that there are many m^6^A modification loci in mRNA of SOCS family members, which implies that the key enzymes of m^6^A modification could regulate mRNA and protein expression of SOCS family members *via* methylation. SOCS1 and SOCS3 have been reported to be aberrantly expressed in some autoimmune diseases and involved in regulating the functions of diverse immune cells (21). Our microarray study also found that SOCS3 was upregulated in the thyroid tissues of GD, implying that SOCS3 and other SOCS family members are related to GD. Recent research showed that METTL3 is involved in liver cancer development by regulating the proliferation and differentiation of liver cells *via* inducing SOCS2 mRNA m^6^A modification (27). Another study found that METTL3 could regulate the differentiation of T cells by inducing SOCS1 and SOCS3 mRNA m^6^A modification (12). We found that METTL3 is downregulated in GD. Therefore, we hypothesized that METTL3 is intensively involved in the pathogenesis of GD by inducing an imbalance of key immune cells. To validate this hypothesis, we knocked down the METTL3 gene in *in vitro* cultured cells to study the influence of METTL3 on GD *via* inducing mRNA m^6^A modification of SOCS family members and its potential molecular mechanisms, which will hopefully provide a novel target for GD therapy. Indeed, we found that mRNA levels of SOCS1, SOCS2, SOCS4, SOCS5, and SOCS6 were increased in METTL3 knockdown cells.

In summary, in this study, we found that METTL3 mRNA declined in GD thyroid tissues using microarray data and functional pathway analysis and further validated the results in clinical PBMCs samples from GD patients. Subsequently, we searched several databases and found that there are multiple mRNA m^6^A modification loci in SOCS family members. At last, we knocked down METTL3 in *in vitro* cultured cells and found that the expression levels of some SOCS family members were enhanced in METTL3 knock-down cells. Overall, the study indicated that METTL3 is involved in the etiology of GD by regulating mRNA levels of SOCS family members including SOCS1, SOCS2, SOCS4, SOCS5, and SOCS6.

## Conclusion

The present study was the first to explore the relationship between m^6^A modification and GD, and revealed that METTL3 may be involved in GD development *via* inducing mRNA m^6^A modification of SOCS family members.

## Data Availability Statement

The original contributions presented in the study are included in the article/supplementary material. Further inquiries can be directed to the corresponding author.

## Ethics Statement

The studies involving human participants were reviewed and approved by the Ethic Committee of Shanghai University of Medicine & Health Sciences Affiliated Zhoupu Hospital (reference number of 2018-C-027-E01). The patients/participants provided their written informed consent to participate in this study.

## Author Contributions

R-hS and J-aZ designed this study. C-qG, PD, and X-rL collected the samples. C-qG extracted RNA from samples. PD performed the real time qPCR. X-rL performed cell culture experiments. R-hS analyzed data and wrote the draft. J-aZ reviewed and revised the manuscript. All authors contributed to the article and approved the submitted version.

## Funding

This work was supported by the National Natural Science Foundation of China (Grant No. 81800696 and 81873636), Science and Technology Development Fund of Pudong New District Minsheng Scientific Research (Medical and Health) Project (No. PKJ2018-Y39), Talent Youth Cultivation Plan of Pudong New District (No. PWRq2020-11), Shanghai Medical Key Specialty (No. ZK2019C09) and “Top-100 Talent Cultivation Plan” of Shanghai University of Medicine and Health Sciences (No. B3-0200-20-311008-30).

## Conflict of Interest

The authors declare that the research was conducted in the absence of any commercial or financial relationships that could be construed as a potential conflict of interest.

## Publisher’s Note

All claims expressed in this article are solely those of the authors and do not necessarily represent those of their affiliated organizations, or those of the publisher, the editors and the reviewers. Any product that may be evaluated in this article, or claim that may be made by its manufacturer, is not guaranteed or endorsed by the publisher.
